# Modifications of traditional pressure gloves for improved performance in scar flexion contracture prevention and fingertip circulation inspection

**DOI:** 10.4103/2321-3868.134083

**Published:** 2014-07-28

**Authors:** Hongliang Zhao, Yan Chen, Cuiping Zhang, Xiaobing Fu

**Affiliations:** 1 General Hospital of People’s Liberation Army, Beijing, China; 2 The First Hospital of Shijiazhuang, Shijiazhuang, Hebei, China; 3 Department of Pharmacy, General Hospital of Beijing Military Region, Dongcheng, Beijing, China; 4 Wound Repair and Tissue Regeneration Laboratory, The First Affiliated Hospital, General Hospital of PLA, 51 Fu Cheng Road, Beijing, 100048 China

Dear editor,

Scar hyperplasia and contracture may occur both during the spontaneous healing process of burn injuries and after surgical correction of burn-injury-related hand dysfunction or deformity such as ulnar claw.[1] Pressure gloves, capable of suppressing local scar hypertrophy and preventing scar contracture formation through scar tissue stretching, are commonly used during the rehabilitation phase in burn patients.[2] Nevertheless, traditional or conventional pressure gloves have significant drawbacks.

**Figure 1:**
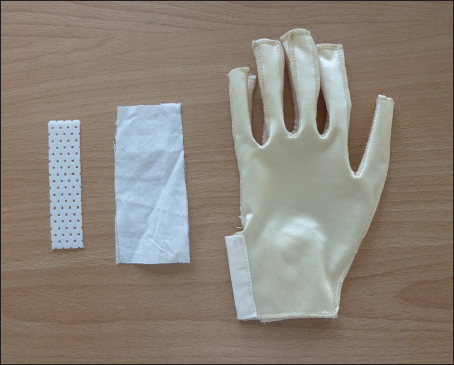
Materials used for an improved pressure glove.

First, traditional gloves are finger closed that cannot adequately keep fingers straight when patients are sleeping or lying in bed. They are also associated with low patient compliance and difficulties being applied to the areas of anatomical flexures because of high-frequency movement. Splints that are designed to stretch tissue scars, prevent scar contractures, and correct the underlying problems may compensate for the inability of traditional pressure gloves to hold fingers in straight positions.[3] However, it is difficult and complicated to fix splints on gloves. To overcome this technical challenge, we attempted to perform a customised modification of the traditional pressure gloves. More specifically, a pocket is generated by sewing a piece of cloth outside the injured finger, allowing for one piece of thermoplastic splints to be inserted in a sandwich manner or two pieces of thermoplastic, one on the top and the other at the bottom of the finger [Figure 1]. This modification enables the use of pressure gloves and splints in combination, which not only has better patient compliance but also allows for adjustments at will to meet the need of movements of the finger with scars.

**Table:** 

Access this article online	
**Quick Response Code**:	**Website**: www.burnstrauma.com
	**DOI**: 10.4103/2321-3868.134083

Another drawback of the conventional finger-closed gloves is the inconvenience in checking up and evaluating the circulation in the distal end of the finger (i.e., the fingertip).[4]

**Figure 2:**
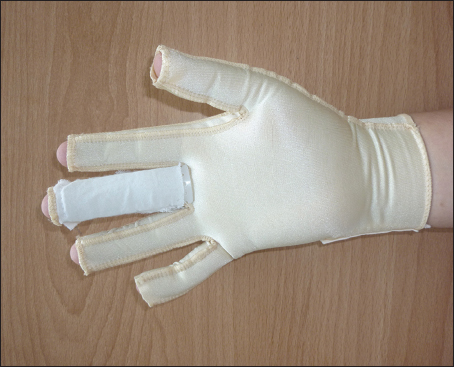
The pressure glove with an additional pocket inserted by a shaped thermoplastic splint and an open window at the distal end of each finger.

To overcome this inconvenience, we made another modification of the traditional pressure gloves by cutting the distal end of each finger open, thereby allowing for easier inspection of the circulation in the fingertip by doctors [Figure 2].
